# Extraction of Peptidoglycan from *L. paracasei subp. Paracasei X12* and Its Preliminary Mechanisms of Inducing Immunogenic Cell Death in HT-29 Cells

**DOI:** 10.3390/ijms160820033

**Published:** 2015-08-24

**Authors:** Pei-Jun Tian, Bao-Long Li, Yu-Juan Shan, Jin-Na Zhang, Jing-Yu Chen, Min Yu, Lan-Wei Zhang

**Affiliations:** 1 School of Food Science and Engineering, Harbin Institute of Technology, No. 73 Huanghe Road, Harbin 150000, China; E-Mails: tianpei0202@gmail.com (P.-J.T.); hgdchenjingyu@gmail.com (J.-Y.C.); 2 Center of Safety and Evaluation of Drugs, Heilongjiang University of Chinese Medicine, No. 24 Heping Road, Harbin 150000, China; E-Mails: lbl73@163.com (B.-L.L.); hzyyumin@gmail.com (M.Y.); 3 School of Municipal and Environmental Engineering, Harbin Institute of Technology, No. 73 Huanghe Road, Harbin 150000, China; E-Mail: zzjjnn-4@163.com

**Keywords:** peptidoglycan, immunogenic cell death, calreticulin, calcium

## Abstract

*L. paracasei subp. paracasei* *X12* was previously isolated from a Chinese traditional fermented cheese with anticancer activities and probiotic potential. Herein, the integral peptidoglycan (X12-PG) was extracted by a modified trichloroacetic acid (TCA) method. X12-PG contained the four representative amino acids Asp, Glu, Ala and Lys, and displayed the similar lysozyme sensitivity, UV-visible scanning spectrum and molecular weight as the peptidoglycan standard. X12-PG could induce the production of apoptotic bodies observed by transmission electron microscopy (TEM). X12-PG could significantly induced the translocation of calreticulin (CRT) and the release of high mobility group box 1 protein (HMGB1), the two notable hallmarks of immunogenic cell death (ICD), with the endoplastic reticulum (ER) damaged and subsequently intracellular [Ca^2+^] elevated. Our findings implied that X12-PG could induce the ICD of HT-29 cells through targeting at the ER. The present results may enlighten the prospect of probiotics in the prevention of colon cancer.

## 1. Introduction

Colon cancer is one of leading causes of cancer death [1]. Several risk factors including westernized diet, obesity and physical inactivity have been demonstrated to closely relate to colon cancer [2]. The microenvironment within colorectal neoplastic lesions significantly differs from the normal, where varieties of bacterial species are enriched such as *Bacteroides fragilis*, *Bacteroides vulgatus*, *Bifidobacterium longum*, *Clostridium butyricum*, *Mitsuokella multiacida*, *Escherichia coli*, *Enterococcus* *faecalis* and *Streptococcus bovis* [3]. The shift from normal microbiota to dysbiosis would increase the pathogenic potential of organisms, ultimately to chronic inflammation and high risks of colon cancer. In 1951, McCoy and Mason reported that a carcinoma of the cecum is associated with enterococcal endocarditis [4]. Since then more and more studies reported that chronic intestinal inflammation is a well-established risk factor for colon cancer [5]. Although the detailed interactions between colonic microflora and colon cancer are not fully understood, a certain composition of gut microbiota may be efficient in the prevention of chronic inflammation and carcinogenesis [6,7].

In recent years, probiotics and prebiotics are increasingly consumed in daily diet. Probiotic has been defined as live microorganisms that confer a number of health benefits on the host, including enhancing immunity, modifying gut associated lymphoid tissue, relieving hypersensitivity, alleviating inflammatory bowel disease, as well as anti-infection and anticancer effects [8], *etc.* Especially probiotic lactobacilli and bifidobacteria are reported to have the capacity of influencing the intestinal microbiota or alleviating certain pathologies involving the gut immune system [9,10]. Other studies suggest that probiotics are protective against colorectal cancer. For example, oral administration of *Lactobacillus acidophilus*-formulated yogurt in mice brought on a significant decrease of colon tumor incidence, tumor multiplicity and tumor size [11]. In addition, a large-scale clinical study on 398 colon cancer patients free from tumor after surgery demonstrated that intake of *Lactobacillus casei* could decline the recurrence rate of colon cancer [12]. Another clinical study showed that oral intake of probiotics could improve the integrity of gut mucosal barrier and the balance of gut microbiota, which contributes to decreasing the infection rate [13]. Moreover, many studies have confirmed that not only the whole probiotics, but also the components and metabolic compositions, distinctively influenced the inhibitory effects on colon cancer [14]. For instance, cell walls of bifidobacterial can stimulate the lymphocytes proliferation and cytokine production [15]. Currently, surgery is still the main therapy for colon cancer but risked a high recurrence rate [16]. The adjuvant therapy such as chemotherapy and radiation therapy inevitably will bring some side effects like myelosuppression and thrombocytopenia [17,18]. Given these, there may be a considerable potential of the application of probiotics in the treatment of colon cancer [19].

In human bodies, the innate immune system is the first defense against the outside attack. This system is built around the germline-encoded pattern recognition receptors, which can recognize certain microbial-associated molecular, and finally trigger the host response. Peptidoglycan, the conserved structural component in the bacterial cell wall, is a good innate immune target [20]. Almost all of the bacteria cell walls contain peptidoglycan; the content of peptidoglycan in Gram-positive bacteria ranges from 30% to 95%, indicating its possible physiological functions. As an activator of the human immune system, peptidoglycan can induce intestinal epithelial cells to produce cytokines such as pro-inflammatory cytokines IL-12p35, IL-8 and tumor necrosis factor-α (TNF-α) in a time-dependent manner [21]. *L. paracasei subp. paracasei X12* (X12) is derived from a traditional cheese in Xinjiang, China. Our previous results showed that the live bacteria inhibited the proliferation of HT-29 cells. Besides, the cell walls extracted from bacteria can also induce apoptosis in HT-29 cells [22]. All these findings implicate the potential of peptidoglycan in the prevention and therapy for colon cancer.

Recent studies suggest that apoptotic cell death could be immunogenic under certain circumstances, which means the tumor cells in early stages of apoptosis would be immunologically recognized, attacked by immunocytes and eventually result in immunogenic apoptosis [23]. The cells undergoing immunogenic death are able to release or translocate specific signals such as calreticulin (CRT), heat shock proteins (HSPs), high mobility group box 1 protein (HMGB1), ATP [24,25,26,27,28], *etc.* Some of the signals are operated on a series of receptors controlled by dendritic cells which present the tumor antigens to T-lymphocytes, constituting a pathway to activate the therapeutic immune responses [29].

Based on the aforementioned information, we hypothesized that the peptidoglycan extracted from X12 (X12-PG) may play an important role in its inhibitory effects on colon cancer [22]. Clarifying the key mechanisms and the target of peptidoglycan will provide novel strategies for exploring, exploiting and applying probiotics products in the future.

## 2. Results

### 2.1. Extraction and Qualitative Analysis of X12-PG

**Figure 1:**
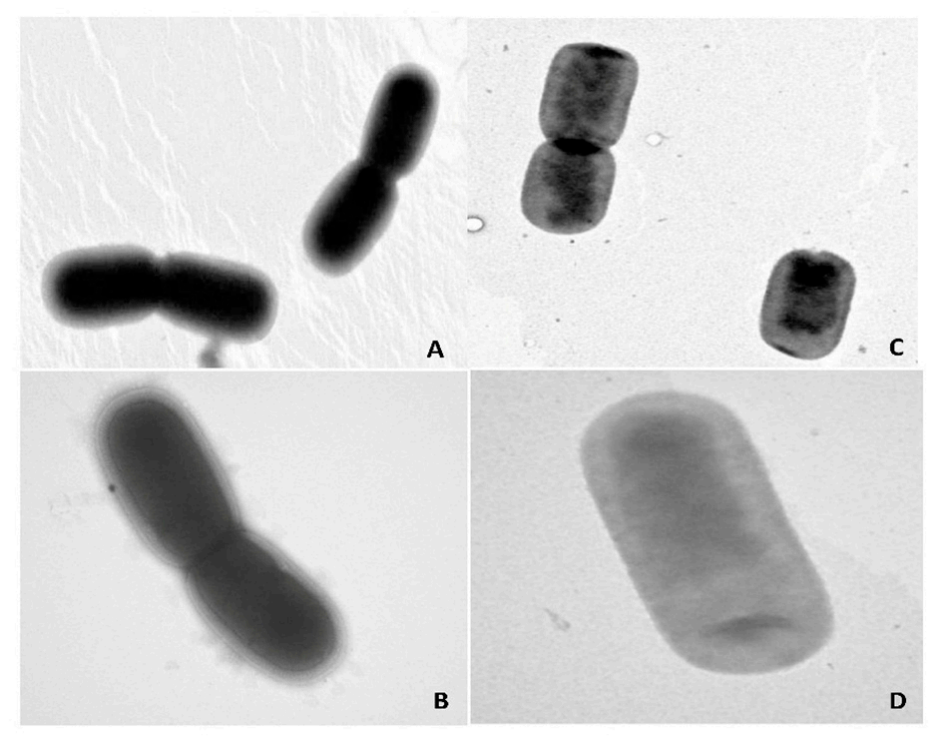
Structural features of the integral peptidoglycan isolated from *L. paracasei subp. paracasei X12* (X12-PG) and the *L. paracasei subp. paracasei X12 bacteria* (X12) observed by transmission electron microscope (TEM). (**A**,**B**) The structure of X12 (×20,000, ×40,000); (**C**,**D**) The cylindrical structure of internal X12-PG (×20,000, ×40,000).

Extraction of X12-PG was processed according to the modified trichloroacetic acid (TCA) method. To maintain the physical integrity of the cell wall structure, this isolation procedure did not result in any physical destructive processes. Also this extraction removed virtually all the teichoic acid from the cell wall. The extraction yield of X12-PG is 6.79%, which was calculated by the dry weight of extracts/bacterial sludge ratio. The results of electron microscopic observation are shown in Figure 1, where a cystic structure and extremely neat edges are seen in X12-PG. In sharp contrast, the X12 bacterial cells are more distinct with dark interior regions.

**Figure 2:**
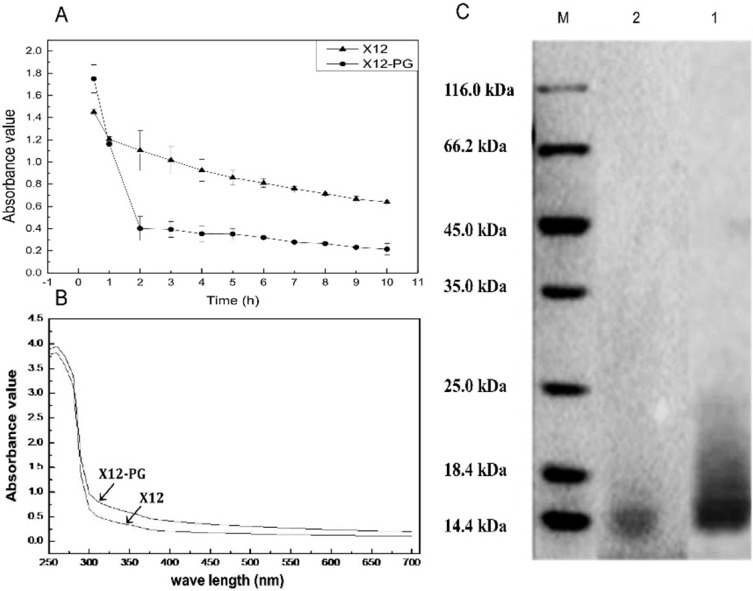
Quantitative and qualitative analysis of X12-PG. (**A**) The degradation models of X12-PG and X12 when exposed to lysozyme. A_450 nm_ of X12-PG declined faster than that of X12; (**B**) The UV-visible scanning spectrum of X12-PG and peptidoglycan standard. The standards and samples were scanned between 190 and 700 nm in a UV/VIS spectrophotometer; and (**C**) The molecular weight (kDa) of X12-PG quantified by SDS-PAGE. The gels were stained with Coomassie Brilliant Blue. The molecular weight was 14 kDa, corresponding to the peptidoglycan standard. Each graph represents the average of more than three replications.

Peptidoglycan is chiefly composed of chains of *N*-acetylglucosamine (GlcNAc) and *N*-acetylmuramic acid (MurNAc) attached to stem peptides. The total carbohydrate content of X12-PG is 199.16 mg/g, the protein content was calculated as 20.31 mg/g. Lysozyme assay is generally used as a qualitative test for peptidoglycan. Because the β-1,4-linked GlcNAc and MurNAc structure in X12-PG can be exclusively hydrolyzed by lysozyme, the absorbance values (A_450 nm_) sharply declined during the first two incubation hours (Figure 2A), whereas no obvious and fast dropping was observed in X12 solution during the whole process. The results suggested that X12-PG possessed the characteristics of peptidoglycan.

Table 1 shows the result of amino acid analysis. Contents of Asp (0.361 mmol/g), Glu (0.381 mmol/g), Ala (0.649 mmol/g), Lys (0.268 mmol/g) were the highest when compared with other amino acids. Moreover, these four amino acids are specifically found in lactobacillus peptidoglycan [30], which in turn revealed that the extracts were essentially peptidoglycan. Evidence to support this belief was supplied by UV-visible scanning spectrum (190–700 nm) and molecular weight of both peptidoglycan standard and X12-PG. They delivered nearly coincident curves with the same absorption peaks and shapes (Figure 2B). The molecular weight of X12-PG was around 14.4 kDa, the same as the standard sample (Figure 2C). Therefore, we confirmed that the extracted X12-PG was peptidoglycan.

**Table 1:** The composition and contents of amino acids.

	Amino Acids	Concentration (μg/mL)	Contents (mmol/g)
1	Glu	56.44	0.381
2	Arg	6.80	0.039
3	Thr	12.74	0.106
4	Tyr	11.11	0.060
5	Val	8.52	0.072
6	Met	11.35	0.076
7	Cys	7.27	0.060
8	Ile	8.19	0.062
9	Phe	9.55	0.057
10	Asp	48.54	0.361
11	Ser	9.75	0.093
12	Gly	7.88	0.105
13	His	12.26	0.078
14	Ala	57.83	0.649
15	Pro	1.02	0.009
16	Leu	16.85	0.128
17	Lys	39.56	0.268

### 2.2. X12-PG Induced Apoptosis in HT-29 Cells

**Figure 3:**
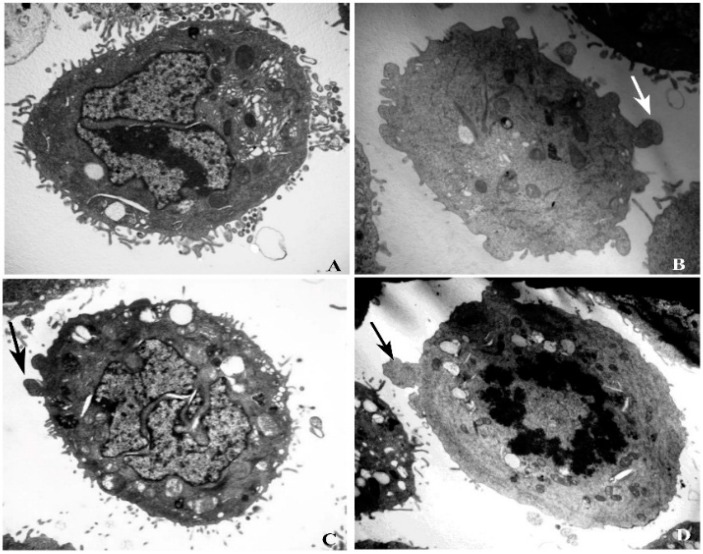
Apoptosis of HT-29 cells after treatment of X12-PG. (**A**–**D**) were respectively treated with 0, 400, 800, 1600 μg/mL X12-PG for 48 h. HT-29 cells were observed by TEM (×6000). The arrows indicate the apoptotic bodies.

Morphological studies of X12-PG-treated and untreated HT-29 cells were carried out by TEM. In untreated cells, a large number of villi-like projections on cell surface were distributed uniformly. In addition, nuclear chromatin and intact organelles such as endoplasmic reticulum, mitochondria and ribosomes, were clearly observed (Figure 3A). In X12-PG-treated cells, however, villous structures were gradually diminished, meanwhile accompanied by mitochondria degradation, increased cytoplasmic vacuoles and formation of apoptotic bodies (Figure 3B,C, arrows showed apoptotic body). This alteration was enhanced when the treatment concentration increased, especially at 1600 μg/mL, villous structures almost completely disappeared. Except for mature apoptotic bodies, cell shrinkage and increased nuclear/cytoplasmic ratio were also observed (Figure 3D).

Peptidoglycan is chiefly composed of chains of *N*-acetylglucosamine (GlcNAc) and *N*-acetylmuramic acid (MurNAc) attached to stem peptides. The total carbohydrate content of X12-PG is 199.16 mg/g, the protein content was calculated as 20.31 mg/g. Lysozyme assay is generally used as a qualitative test for peptidoglycan. Because the β-1,4-linked GlcNAc and MurNAc structure in X12-PG can be exclusively hydrolyzed by lysozyme, the absorbance values (A_450 nm_) sharply declined during the first two incubation hours (Figure 2A), whereas no obvious and fast dropping was observed in X12 solution during the whole process. The results suggested that X12-PG possessed the characteristics of peptidoglycan.

The effects of X12-PG on cell proliferation were measured by MTT assay. As shown in Table 2, the inhibition rate of HT-29 cells became more significant when treatment concentration increased. We used oxaliplatin (OXP) as a standard ICD inducer that is widely approved [31], and which result in an inhibition rate of 74.21%. In our previous article we reported the minor toxic activity of X12-PG in noncancerous cells when compared with that in HT-29 cells; they released selective growth inhibitors, which specifically targeted cancer cells [22], so X12-PG can be investigated in a safe anticancer therapy agent. After treatment with 1600 μg/mL X12-PG, the apoptotic cells (%) reached 61.03%.

**Table 2:** Cell viability measured by MTT assay.

Treatment	Apoptotic Cells (%)
OXP (15 μmol/L)	74.21 ± 0.38
X12-PG (400 μg/mL)	50.42 ± 0.46
X12-PG (800 μg/mL)	55.65 ± 0.40
X12-PG (1600 μg/mL)	61.03 ± 0.35

### 2.3. Damaged to Endoplasmic Reticulum Induced by X12-PG in HT-29 Cells

**Figure 4:**
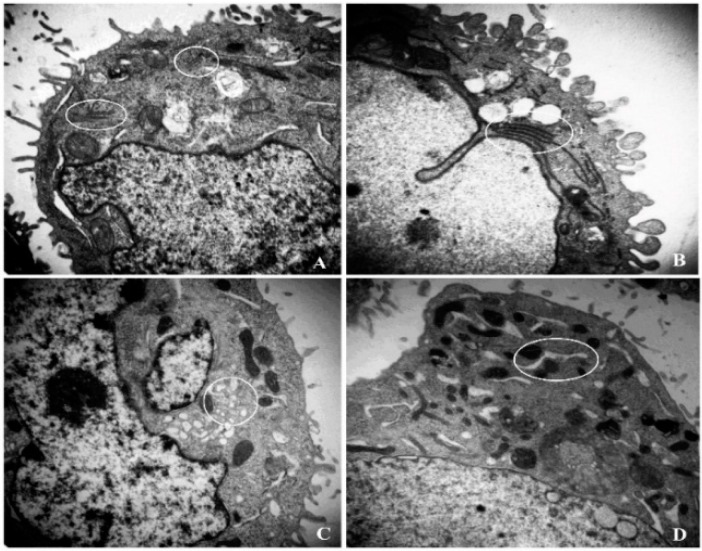
Structural damages of endoplasmic reticulum (ER) after treatment of X12-PG. (**A**–**D**) were respectively treated with 0, 400, 800, 1600 μg/mL X12-PG for 48 h. Cells were observed by TEM (×20,000). The encircled regions show the location of ER.

The endoplasmic reticulum (ER) is an important organelle that fulfills a multiple of cellular functions. In most cases, cytoplasmic ER morphologically shapes like thin lines (Figure 4A). Compared with the control, when treated with 400 μg/mL X12-PG, ER became darker and swelled. Moreover, hair-like projections on the membrane were gradually distorted and reduced (Figure 4B). X12-PG of 800 μg/mL led to the formation of vacuoles and clearer ER dilation (Figure 4C). At the dose of 1600 μg/mL, the ER were further swelled in larger quantities (Figure 4D). These results suggest that X12-PG was able to alter structural features of ER and eventually damage its physiological function.

### 2.4. X12-PG Induced the Translocation of Calreticulin (CRT) and Release of HMGB1

**Figure 5:**
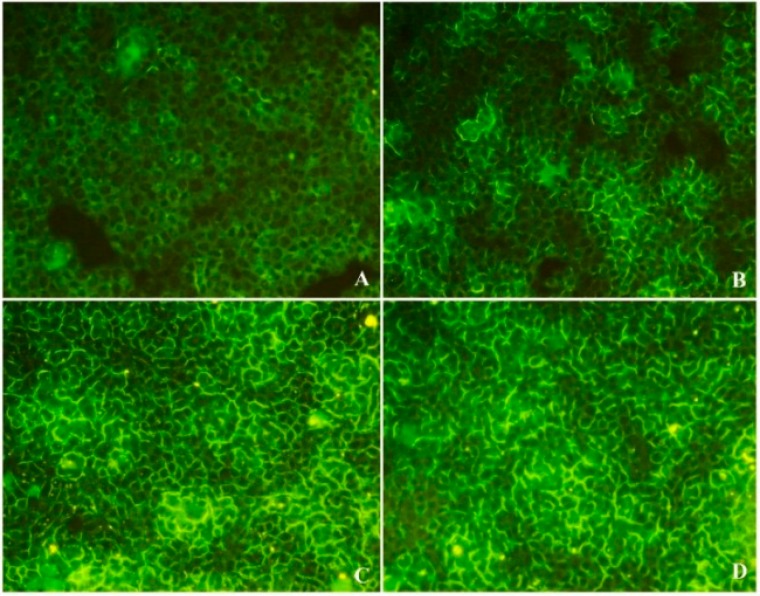
Calreticulin (CRT) exposure detected by immunofluoroscence assay after treatment of X12-PG. (**A**–**D**) were respectively treated with 0, 400, 800, 1600 μg/mL X12-PG for 48 h, then incubated with primary antibody (Anti-Calreticulin, diluted 1:200 in blocking buffer) and detected with the FITC-labeled secondary antibody. Fluorescence intensity was visualized by immunofluorescence microscopy (×200).

**Figure 6:**
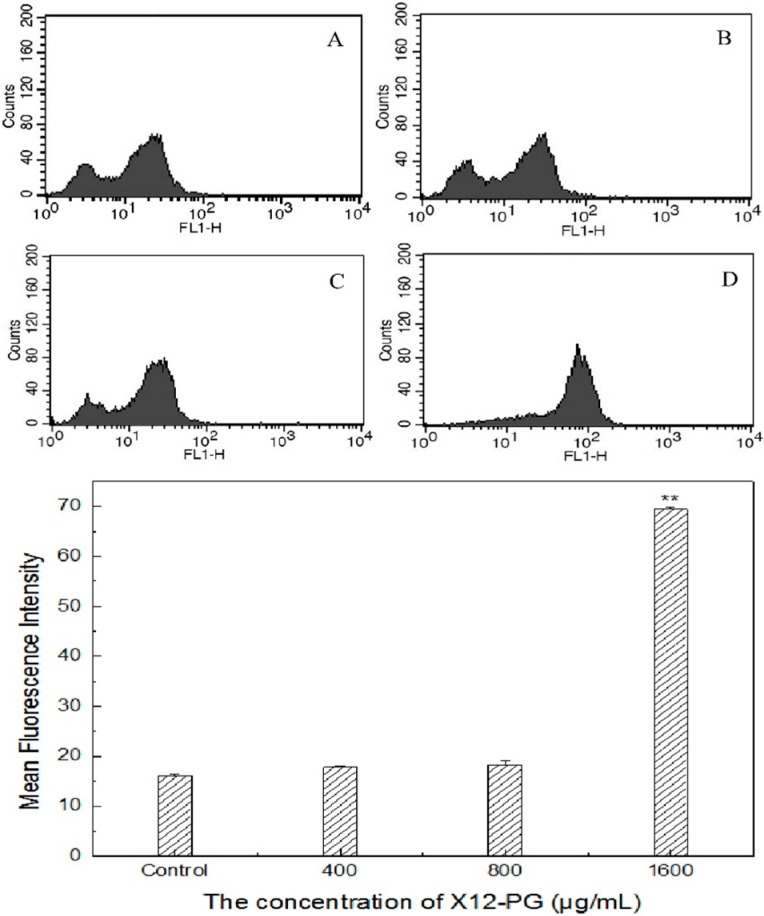
CRT exposure detected by flow cytometry after treatment of X12-PG. Prior to analysis, cells were respectively treated with 0, 400, 800, 1600 μg/mL (**A**–**D**) X12-PG for 48 h, then harvested and labeled with FITC. The concentration of CRT on cell membrane was expressed as mean fluorescence intensity (MFI) which has excluded background (Isotype-matched IgG was used as a control: Data not shown). (Error bars are mean ± SD, ** *p* < 0.01 *versus* control)

CRT is a high-affinity Ca^2+^-binding protein. The majority of cellular CRT is located in the ER, where it participates in modulating intracellular Ca^2+^ homeostasis and Ca^2+^ signaling. After stimulation (48 h) for X12-PG, CRT was exposed on the cell surface, as determined by immunofluorescence staining (Figure 5) or flow cytometry analysis (Figure 6). Sparse but clear fluorescence (Anti-CRT-FITC) on the membrane emerged when treated with 400 μg/mL X12-PG. Fluorescence intensity was enhanced when treated with a range of increasing concentrations, indicating more and more CRT translocated to the cell membrane (Figure 5). In identical conditions, the content of CRT was quantified by flow cytometry based on mean fluorescence intensity (MFI): CRT exposure significantly (*p* < 0.01) increased when treated with 1600 μg/mL X12-PG (Figure 6).

Dying cells also released the cell death–associated nuclear HMGB1 protein into the extracellular milieu in a dose dependent manner (Table 3). When stimulated with 800 and 1600 μg/mL X12-PG, the concentration of HMGB1 in the supernatants were increased by 109% (15.86 ± 0.32 ng/mL, * *p* < 0.05) and 185% (21.58 ± 0.36 ng/mL,** *p* < 0.01) respectively compared with the control (7.56 ± 8.5 ng/mL). Altogether, these results revealed the ability of X12-PG in eliciting CRT translocation and HMGB1 secretion, which are necessary for signaling immunogenicity and ultimately leading to ICD.

**Table 3:** ELISA detection of HMGB1 release in the supernatants of HT-29 cells.

X12-PG Treatment (μg/mL)	HMGB1 Concentration (ng/mL)
0	7.56 ± 0.35
400	9.52 ± 0.35
800	15.86 ± 0.32 *
1600	21.58 ± 0.36 **

### 2.5. X12-PG Promoted the Release of Ca^2+^ from the ER into the Cytoplasm

**Figure 7:**
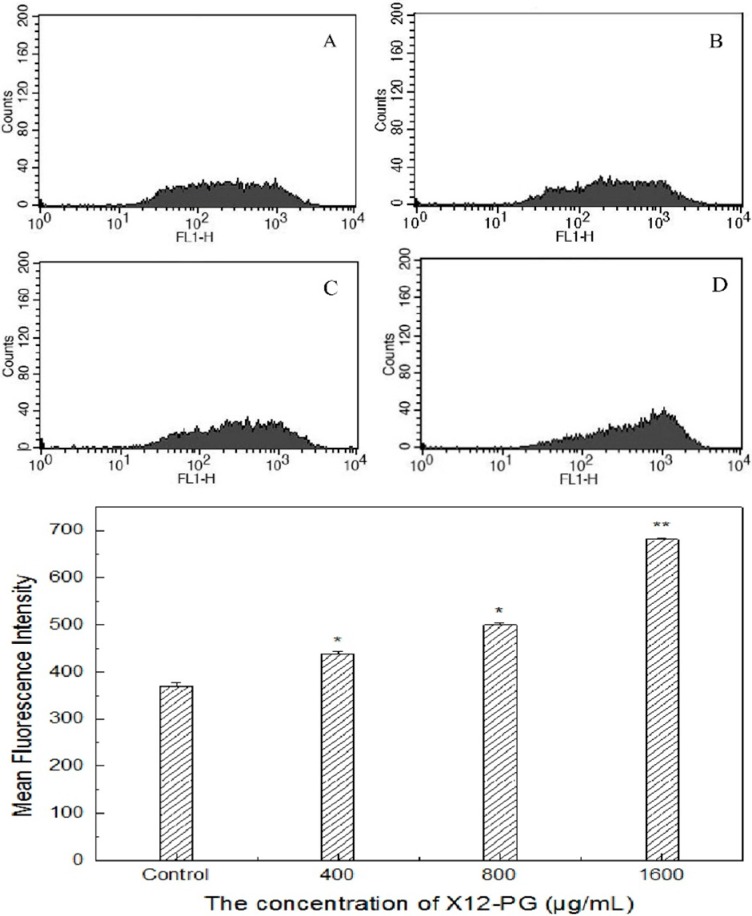
Intracellular Ca^2+^ concentration ([Ca^2+^]) after treatment of X12-PG. (**A**–**D**) were respectively treated with 0, 400, 800, 1600 μg/mL X12-PG for 48 h. Cytosolic [Ca^2+^] was increased in a dose-dependent manner after exposure to X12-PG. HT-29 cells were loaded with Fluo-3/AM and analyzed by flow cytometry. Fluo-3/AM was excited at the 488 nm line of an argon laser and the fluorescence intensity was measured at an emission wavelength 530 nm. [Ca^2+^] was expressed as mean fluorescence intensity (MFI). (Error bars are mean ± SD, * *p* < 0.05 *versus* control, ** *p* < 0.01 *versus* control).

We next examined the correlation among cytosolic free Ca^2+^, CRT exposure and ER dysfunction. Normally, Ca^2+^ is stored in the ER where physiological activities are regulated through the uptake or release of Ca^2+^. Intracellular Ca^2+^ concentration ([Ca^2+^]) was measured with the fluorescent probe Fluo-3/AM by flow cytometry. The MFI of [Ca^2+^] in the cytoplasm were increased by 18% (437.5 ± 6.5), 35% (499.5 ± 4.5) and 85% (681.5 ± 3.5, *p* < 0.01) respectively, compared with the control group (369.5 ± 8.5) after exposure to 400, 800, 1600 μg/mL X12-PG for 48 h (Figure 7). Before this, we had observed X12-PG-induced structural damage of ER, as well as CRT translocation. Although the mechanisms accounting for CRT exposure remain largely elusive, based on the data summarized in this work, we assumed that ER [Ca^2+^] homeostasis plays a major role in the regulation of CRT exposure as it is induced by X12-PG.

## 3. Discussion

In the present study, we extracted peptidoglycan (X12-PG) from the *L.paracasei subp. paracasei X12* which previously showed potential anti-colon tumor activities. The current results revealed that X12-PG could induce immunogenic apoptosis in HT-29 cells, characteristically displayed by CRT translocation, HMGB1 secretion, the increased intracellular [Ca^2+^] levels, as well as some classical evidence, for instance, apoptotic bodies and ER damage.

For the compact structure of bacterial cell wall, it is difficult to find a proper isolation method that can remove all the cell contents but concurrently retain structural integrity of the cell wall. The earliest method of integral peptidoglycan extraction is documented by Sekine *et al.* [32]. According to this method, Yue and Hu successfully extracted the integral peptidoglycan from *Bifidobacterium* sp. [33], however, the extraction step is complicated, with a long extraction period but low extraction yield. Herein, we slightly modified the TCA method in order to shorten the extraction period and improve the yield. The yield reached 6.79% in a four-day extraction. Quantitative and qualitative determinations such as lysozyme sensitivity [34], molecular weight and amino acid analysis further confirmed that X12-PG was essentially a peptidoglycan [35].

It is generally assumed that eradicating all cancer cells-including cancer stem cells and early micrometastases-by direct cytostatic or cytotoxic effects, contributes to the complete and permanent cure of cancer. Based on this simplistic belief, induction of apoptosis by conventional chemotherapy or radiotherapy is thought to be non-inflammatory and non-immunogenic. Accumulated evidence has demonstrated that long-term clinical success of chemotherapeutics, such as anthracyclines- or oxaliplatin-induced apoptotic cell death, can be immunogenic and hence elicit active immune responses against dying tumor cells [36]; this mechanism is commonly known as immunogenic cell death (ICD).

Nonetheless, chemotherapy is still the most common and effective treatment for various cancers, it should be noted that the side effect of cytotoxic and new oncological danger would impact the quality of life from these treatments [37]. As already known, a large number of probiotic lactobacillus exerts potential anticancer effects by inducing apoptosis or promoting the production of immune factors. While the underlying mechanisms require further exploration, not only the whole live cells, but also the heat-killed cells, cell walls and cytoplasm exhibited the abilities of suppressing the growth of colon cancer cells [38,39,40]. Interestingly, the X12-PG extracted from *L. paracasei subp. paracasei X12* could induce cell apoptosis and CRT exposure in HT-29 cells. Driven by these considerations, we established the primary mechanism of X12-PG-induced ICD in HT-29 cells and the anticancer potential of probiotics.

**Figure 8:**
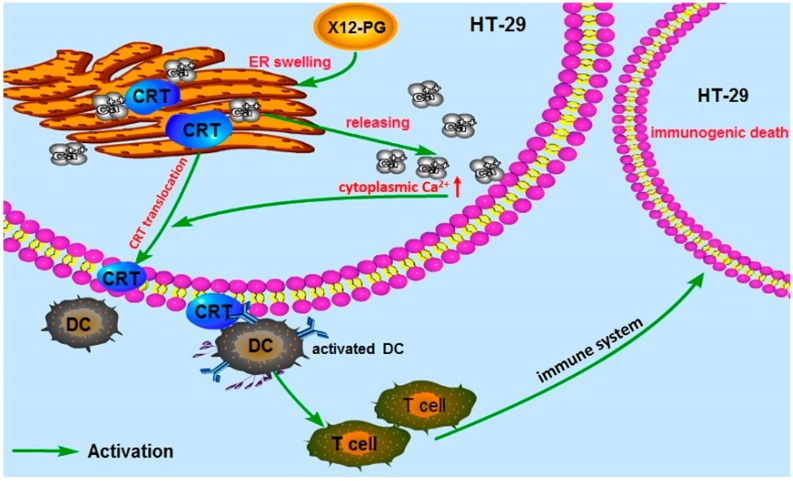
Properties of immunogenic cell death (ICD) induced by X12-PG in HT-29 cells. As a result of endoplasmic reticulum stress (stimulated by X12-PG), cancer cells expose CRT on their plasma membrane at a pre-apoptotic stage. This facilitates the recruitment of dendritic cells (DCs) into the tumor bed, the engulfment of tumor antigens by DCs (stimulated by CRT), and optimal antigen presentation to T cells (stimulated by HMGB1). CRT served as an “eat-me” signal, and CRT exposure may be regulated by cytosolic [Ca^2+^].

CRT exposure on the membrane serves as an engulfment signal in pre-apoptotic cells for specific interaction with dendritic cells (DCs), which could engulf, process, and present antigen from dying tumor cells to T-lymphocytes. The activated immune defensing system will eventually kill the tumor cells. Obeid *et al.* reported that CRT was exposed on the surface of cells undergoing ICD, but was lacking in those cells with non-immunogenic cell death [24]. Conversely, anti-neoplastic agents that fail to induce CRT exposure are intrinsically incapable of provoking ICD [41]. All these studies identify CRT as a major checkpoint of ICD. CRT is an abundant chaperone that is normally secluded in the ER lumen; it is able to translocate to the plasma membrane under a series of ER stress responses through a complex pathway, including paracrine signals and transduction cascade activations. CRT is also a high capacity Ca^2+^-binding protein of intracellular Ca^2+^ stores and CRT translocation breaks ER-regulation of Ca^2+^ homeostasis [42]. Moreover, this disturbance may synergistically aggravate CRT translocation. As described in a previous study, after cells were transfected with a Reticulon-1C (an evolutionary conserved protein to decrease the [Ca^2+^] in the ER) overexpression plasmid, the ER could no longer mediate CRT exposure in response to anthracyclin treatment [43]. In addition, cells undergoing ICD release the nuclear protein HMGB1 as their membranes become permeabilized during secondary necrosis [29]. HMGB1 can interact with several receptors expressed on the surface of DCs including the toll-like receptor 4 (TLR4) [26,28]. Combing the previous studies and our experimental results, we speculate that the ER structure damages induced by X12-PG treatment, directly results in the reduction of endoplasmic reticulum [Ca^2+^] levels and elevation of intracellular [Ca^2+^]. Subsequently, Ca^2+^ served as a translocation signal and triggered CRT exposure onto the cell surface (Figure 8). To our knowledge, no detailed molecular mechanisms of how CRT translocated to the cell membrane has been clearly clarified, and it is a problem that requires further investigation.

## 4. Experimental Section

### 4.1. Reagents

Peptidoglycan standard, Anti-Calreticulin, MTT, were obtained from Sigma Co. (St. Louis, MO, USA). Bradford Protein kit, Fulo-3/AM and Fluorescein Isothiocyanate (FITC)-labeled secondary antibody (goat anti-rabbit) were from Bi YunTian biotechnology Co., Ltd. (Shanghai, China). Coomassie brilliant blue G250 and DMSO were purchased from Lianxing Biotechnology Co., Ltd. (Beijing, China). Other reagents were all analytical grade and supplied by Sinopharm Chemical Reagent Co., Ltd. (Beijing, China).

### 4.2. Cell Culture

HT-29 cells (human colon adenocarcinoma cell line), provided by the Cancer Institute of the Chinese Academy of Medical Science (Beijing, China), were cultured in RPMI-1640 medium supplemented with 10% fetal-bovine serum (FBS), 1% l-glutamine, penicillin (100 units/mL) and streptomycin (100 µg/mL). Cells were maintained at 37 °C in a humidified atmosphere with 5% CO_2_.

### 4.3. Isolation and Purification of X12-PG

*L. paracasei subp. paracasei X12* strain was separated from a traditional fermented cheese in Xinjiang province, China. The live bacteria had been previously demonstrated to exert anti-proliferative activity and highly adhering capability on HT-29 cells [22]. *L. paracasei subp. paracasei X12* was cultured in de Man, Rogosa, Sharpe (MRS) (Difco, Detroit, MI, USA) broth with 0.05% l-cysteine at 37 °C under anaerobic conditions. The strain was identified by 16S rRNA gene sequences and sub-cultured twice at 37 °C for 18 h before use.

The extraction of X12-PG is based on the TCA method as described by Sekine *et al.* [32]. Briefly, bacterial sludge was washed in 0.9% saline solution (*w*/*v* = 1:10) until it turned white. The clean sludge was dissolved in 10% TCA (*w*/*v* = 1:10), incubated in a boiling bath for 60 min, and then centrifuged at 20,000× *g* for 20 min. Collecting the sediment and treated with a special solvent (Acetic acid-sodium acetate buffer (using 0.5 M acetic acid and 0.2 M sodium acetate, pH 4.5) chloroform, methanol mix with the ratio of 4:5:10 (*v*/*v*/*v*), pH 4.6) overnight. After centrifugation (1000× *g* for 10 min), the insoluble residue was incubated in Tris-HCL (0.1 M, pH 7.5) containing 2000 U trypsin at 37 °C in a shaking bath (140 rpm) for 12 h. Finally, the mixture was centrifuged at 20,000× *g* for 20 min. The sediment was harvested and washed in sterile water 3–4 times, then lyophilized and stored in an airtight container at −20 °C for further analysis.

### 4.4. Analytical Methods of X12-PG

For determination of the β-1,4-linked MurNAc and GlcNAc, X12-PG was treated with lysozyme (200 μg/mL, dissolved in 1 mg/mL PBS, pH 6.2) at 37 °C in a shaking bath (140 rpm). X12 was treated identically for comparison. Collecting the reactant every hour, measured its UV absorbance at 450 nm in a UV/VIS spectrophotometer (METASH UV-5100; Metash: Shanghai, China) during the reaction [34].

The molecular weight of X12-PG was determined by SDS-PAGE analysis. Electrophoresis was carried out in 30% polyacrylamide gels. The gels were then stained with Coomassie Brilliant Blue G250 and the molecular weight was determined by comparing with the band location of peptidoglycan standard.

For quantitative amino acid analyses, X12-PG samples were hydrolyzed by 6 M HCl (*v*/*v* = 1:9) in a nitrogen atmosphere at 110 °C for 22 h. The solution was filtered through a 0.45 μm membrane filter and quantitative analyses were performed in an amino acid analyzer (Hitachi L-8800; Hitachi: Ibaraki, Japan).

Total carbohydrate content was measured by Phenol-sulfuric acid method. The standard procedure of this method was documented previously [44]. The standard curve was mapped based on the regression equation with a coefficient ≥0.999 (*Y* = 0.00902*X* + 0.00837, *R*^2^ = 0.9993). The protein content in X12-PG was measured using a Bradford Protein kit according to the manufacturer’s instructions. (*Y* = 1.77705*X* + 0.02152, *R*^2^ = 0.9992).

### 4.5. Transmission Electron Microscope (TEM)

The HT-29 cells were seeded in a 6-well plate (1 × 10^6^ cells/mL) and treated with 0, 400, 800, 1600 μg/mL X12-PG for 48 h. The cell pellets were harvested and re-suspended in melted agar medium (2.5%). The cooled solidified medium was cut into small blocks (5 × 2 × 1 mm), fixed with 5% (*v*/*v*) glutaraldehyde in PBS (0.1 M, pH 7.3) for 2 h, then in 2% (*w*/*v*) osmic acid with the same buffer for 2 h. The blocks were further processed for dehydration, infiltration in a graded ethanol series as previously described [45]. Ultrathin sections were treated with saturated uranyl acetate for 20 min, counterstained with 0.3% lead citrate for another 20 min, finally examined with a Hitachi H-7650 transmission electron microscope in Harbin Medical University.

### 4.6. MTT Assay

HT-29 cells were plated into 96-well microtiter plates (1 × 10^6^ cells/mL) and treated with 0, 400, 800, 1600 μg/mL X12-PG for 48 h. MTT (3-(4,5-dimethylthiazol-2-yl)-2,5-diphenyltetrazolium bromide) stock solution (5 mg/mL) at 10 μL per well was added for an additional 4 h. The supernatant was aspirated and the formazan crystals were solubilized in dimethylsulfoxide, followed by determination of optical densities at 560 and 690 nm using a microtiter plate reader. The spectrophotometer was calibrated to zero absorbance using culture medium without cells. All experiments were run eight times.

### 4.7. Immunofluoroscence Assay

Immunofluoroscence assay was performed as previously described [22,46]. The HT-29 cells were cultured in a 6-well plate (1 × 10^6^ cells/mL), after treated with 0, 400, 800, 1600 μg/mL for 48 h, the cell pellets were fixed with 4% paraformaldehyde on ice for 20 min and treated with a blocking buffer (1% bovine serum albumin and 1% fish gelatin in PBS, pH 7.3) at 37 °C for 30 min. The cells were incubated with primary antibody (Anti-Calreticulin, diluted 1:200 in blocking buffer) and detected with the FITC-labeled secondary antibody. CRT exposure on cell membranes were visualized by immunofluorescence microscopy (Olympus BX51; Olympus Optical: Tokyo, Japan) and photographed.

### 4.8. Flow Cytometry Assay

For measuring alterations of intracellular [Ca^2+^] after X12-PG treatment, HT-29 cells (1 × 10^6^ cells/mL) were loaded with Ca^2+^ indicator dye by incubating in a Fluo-3/AM buffer (5 μM Fluo-3/AM and 25% (*w*/*w*) pluronic was mixed in dry DMSO) for 30 min at room temperature [47]. The cells were diluted to 1 × 10^6^ cells/mL in PBS and incubated for 10 min at 37 °C to equilibrate calcium stores, then analyzed by flow cytometry (Becton–Dickinson: East Rutherford, NJ, USA). Fluo-3/AM was excited at the 488 nm line of an argon laser and the fluorescence intensity was measured at an emission wavelength 530 nm. The determination of CRT translocation onto cell membrane was performed similarly, except that HT-29 cells were stained with FITC as described in 4.7. Isotype-matched IgG antibodies were used as a control.

### 4.9. Detection of HMGB1 Release

HT-29 cells were plated in 6-well plates (1 × 10^6^ cells/mL) with 2 mL medium. The medium was changed 24 h later and treatment was added into the cells. Supernatants were collected by centrifugation and were frozen immediately. Quantification of HMGB1 in the supernatants was assessed by enzyme-linked immunosorbent assay according to the manufacturer’s instructions (*Y* = 0.00706*X* + 0.00467, *R*^2^ = 0.9935).

### 4.10. Statistical Analysis

All experiments were performed in triplicate and results were expressed as means ± standard deviation (SD). Statistical analyses were carried out with SPSS 19.0 (SPSS Inc.: Chicago, IL, USA). One-way ANOVA followed by Duncan’s post hoc test were used. Results were considered statistically significant when *p* < 0.05 and highly significant when *p* < 0.01.

## 5. Conclusions

In this study, we extracted X12-PG from the *L.paracasei subp. paracasei X12* using a modified TCA method. X12-PG contained the four representative amino acids Asp, Glu, Ala and Lys, and displayed similar lysozyme sensitivity, UV-visible scanning spectrum and molecular weight as the peptidoglycan standard, all of which indicated that X12-PG was essentially a peptidoglycan. X12-PG can also induce ICD in HT-29 cells, with the hallmarks of CRT exposure and through the ER-targeted as well as Ca^2+^-signaling pathway. To our knowledge, few studies have reported the immunogenic induction mediated by probiotics. Our studies further enlighten the future application of probiotics as a dietary anticancer therapy.
